# Cataract‐Causing Mutant R188C of βB2 Crystallin With Low Structural Stability is Sensitive to Environmental Stresses and Prone to Aggregates Formation

**DOI:** 10.1002/EXP.20240192

**Published:** 2025-04-01

**Authors:** Yibo Yu, Silong Chen, Ying Zhang, Hang Song, Jiarui Guo, Chengpeng Wu, Wei Wu, Jingjie Xu, Xiaoyu Cheng, Chenqi Luo, Jing Guo, Yip Chee Chew, Ke Yao, Xiangjun Chen, Lidan Hu

**Affiliations:** ^1^ Eye Center of Second Affiliated Hospital, School of Medicine Zhejiang University Hangzhou China; ^2^ Institute of Translational Medicine Zhejiang University School of Medicine Hangzhou China; ^3^ Department of Ophthalmology Peking Union Medical College Hospital Chinese Academy of Medical Sciences and Peking Union Medical College Beijing China; ^4^ Centre for Computational Biology (CCB) Duke‐NUS Medical School, 8 College Road Singapore Singapore; ^5^ Ophthalmology and Visual Sciences Department Khoo Teck Puat Hospital Singapore Singapore; ^6^ National Clinical Research Center for Child Health The Children's Hospital Zhejiang University School of Medicine Hangzhou China

**Keywords:** aggregates, cataract, structural stability

## Abstract

This study investigated a Chinese family with congenital posterior polar cataracts linked to the βB2‐R188C mutation. βB2‐crystallin, a key structural component of the lens, is crucial for maintaining lens transparency and stability. We examined the effects of the R188C mutation on βB2‐crystallin's structural stability and resistance to environmental stressors using purified proteins and cellular models. The βB2‐R188C mutant showed poor stability and a tendency to aggregate under physiological and pathological conditions. The mutation disrupted the oligomerization equilibrium, causing dissociation of dimers into monomers. Molecular dynamics simulations and spectroscopic experiments revealed abnormal protein folding induced by the R188C mutation, increasing susceptibility to environmental stressors. Aggregation was observed in both prokaryotic and eukaryotic models under normal conditions, with enhanced severity under environmental stressors. Notably, lanosterol treatment or αB‐crystallin partially reversed aggregation. In summary, the R188C mutation promotes abnormal aggregation by destabilizing βB2‐crystallin and disrupting oligomerization equilibrium, potentially leading to cataract formation. Targeting aggregate formation with small molecules like lanosterol or enhancing molecular chaperone activity offers a promising strategy for cataract prevention and treatment.

## Graphical abstract

This study investigated the role of the βB2‐crystallin c.562C>T (p.R188C) mutation in congenital cataract. Clinical pedigree analysis, combined with whole‐exome sequencing (WES), identified this pathogenic variant. Bioinformatic analyses predicted destabilization of the mutant protein’s tertiary structure, potentially impairing its function. In vitro experiments demonstrated that the mutant protein formed aggregates under cellular stress, which were effectively mitigated by treatment with lanosterol and αB‐crystallin. By integrating clinical observations, computational modeling, and experimental validation, this work elucidates how the mutation drives cataract pathogenesis and provides mechanistic insights for pharmacological intervention. These findings suggest a novel strategy for developing non‐surgical therapies targeting protein aggregation in congenital cataracts.

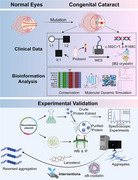

## 1 Introduction

Cataract is a disease marked by lens opacity, mainly caused by unusual interactions between genetic and environmental factors with the lens proteins [1, 2]. Cataracts are classified into congenital and acquired types depending on their pathogenesis. Congenital cataracts, which are the primary global cause of childhood blindness, arise from diverse factors, with genetic mutations serving as the most significant contributor [3, 4, 5]. To date, over 100 gene mutations associated with congenital cataracts have been identified, affecting a variety of proteins. These include crystallins such as αA‐crystallin (CRYA), αB‐crystallin (CRYαB), and βB‐crystallin (CRYβB2). Mutations in membrane proteins like macrophage inflammatory protein (MIP) and gap junction protein (GJA) have also been implicated. Additionally, cytoskeletal proteins and transcription factors, including Forkhead box protein E3 (FOXE3) and heat shock transcription factor 4 (HSF4), are associated with congenital cataracts [6, 7]. Among these, mutations in crystallins account for more than half of the reported cases, and the pathogenesis of some mutations remains unclear.

Crystallins constitute the predominant soluble proteins within the vertebrate lens and play a critical role in preserving lens transparency [8]. These proteins are divided into three primary categories based on their relative abundance: α‐crystallin (accounting for 40%), β‐crystallin (35%), and γ‐crystallin (25%) [9]. α‐crystallin, a molecular chaperone and member of the heat shock protein family, is essential for preserving protein stability and ensuring lens clarity [10]. β‐ and γ‐crystallins, both structural proteins, possess similar tertiary structures and are defined by the presence of four highly conserved Greek key motifs. The presence of these motifs is essential for preserving the structural integrity and facilitating appropriate protein interactions within the lens [11, 12, 13]. While γ‐crystallin predominantly exists as monomers, β‐crystallin has the capacity to form multiple oligomers (from 2 to 8 mer). Based on their acid–base properties, β‐crystallins can be classified into two subtypes: acidic βA‐crystallin and alkaline βB‐crystallin. The physiological function of β/γ‐crystallins is dependent on their structural stability. Mutations in the Greek key motifs can disrupt the three‐dimensional conformation, leading to protein aggregation and denaturation, which are common features of cataract formation [14, 15].

Among the β‐crystallins, βB2‐crystallin is an alkaline protein, recognized as the most stable and abundant isoform, playing a key role in lens transparency and stability [16]. The βB2‐crystallin structure consists of two compact domains that are connected by a linker peptide, along with extensions at both the N‐terminus and C‐terminus. Each domain consists of four β‐sheets forming a Greek key motif [17]. βB2‐crystallin can form homodimers, heterodimers, and other oligomers, typically presenting as a stable face‐to‐face dimeric structure in solution. The formation of aggregates is associated with the unfolding of its conformation [18]. The Cat‐Map database (https://cat‐map.wustl.edu/) documents numerous cases of congenital cataracts linked to βB2‐crystallin mutations. Conversely, most research has been limited to case reports, leaving the pathogenesis of βB2‐crystallin mutants barely explored. According to the latest version of the database in October 2024, 30% of crystallin‐related congenital cataracts are βB2‐crystallin mutations, and five of the 126 reported βB2‐crystallin proband mutations are at position 188. In this study, we investigated a cataract‐related mutation in *βB2‐crystallin* (c.562C > T, p.R188C), which was initially identified in our hospital. Our objective was to gain further insights into the aggregation regulation and pathogenic mechanism of the βB2‐R188C mutation by purifying wild type βB2‐crystallin and the βB2‐R188C mutant crystallin. We aimed to evaluate how the R188C mutation affects the structural stability of βB2‐crystallin and discovered that lanosterol effectively mitigates the formation of pathogenic aggregates.

## 2 Methods and Materials

### 2.1 Plasmid Preparation

The *βB2‐crystallin* template sequence stored in our laboratory was amplified using polymerase chain reaction (PCR) and compared with the *βB2‐crystallin* sequence (NM_00496.3) obtained through NCBI (The National Center for Biotechnology Information) by second‐generation sequencing. Subsequently, the *CRYβB2‐R188C* mutant sequence was generated through site‐directed mutagenesis. Both the wild‐type and mutant genes were then cloned into the pET‐28a and pEGFP‐N1 vectors using standard molecular cloning techniques and *αB‐crystallin* gene was cloned into mCherry‐C1 vectors. All recombinant plasmids were validated by DNA sequencing [19].

### 2.2 Techniques for Protein Extraction and Purification

Recombinant proteins were produced in *Escherichia coli* Rosetta (DE3) cells  cultured in an LB medium containing 50 µg mL^−1^ kanamycin. Protein expression was induced  by adding 0.1 mM IPTG (Sangon Biotech, Shanghai, China) at 16°C for 16 h. The harvested bacterial strains were resuspended in lysis buffer (20 mM Na₂HPO₄, 150 mM NaCl, 1% PMSF, pH = 7.4) and lysed via ultrasonication at 4°C. The lysate supernatant was then subjected to purification using Ni‐affinity chromatography. Nonspecifically bound proteins were removed with a wash buffer containing 60 mM imidazole, while the target protein was eluted with 500 mM imidazole. Further purification was achieved using size‐exclusion chromatography (SEC) on a Hiload 16/60 Superdex 200 column, employing an ÄKTA pure chromatography system (Cytiva, Sweden) with an elution buffer (150 mM NaCl, 20 mM Na₂HPO₄, 1 mM EDTA, pH = 7.2). The purity of the protein (>95%) was confirmed by Coomassie blue staining.

### 2.3 Spectroscopy

Before conducting protein spectroscopy experiments, all protein concentrations were dissolved and adjusted to 10 and 50 µM by SEC buffer. All experiments were conducted using a 10 mm optical path colorimetric dish.

Protein fluorescence, both endogenous and extrinsic, was evaluated using a spectrofluorometer (Hitachi Ltd., Tokyo, Japan). Tryptophan fluorescence was monitored at an excitation wavelength of 295 and 280 nm, while ANS fluorescence was detected at 380 nm. For ANS fluorescence assays, the protein solution was combined with the ANS probe (Sigma‐Aldrich) at a ratio of 1:100 before measurement. Turbidity of the protein solution was measured by absorbance at 400 nm (A_400_) using an Evolution 300 Security UV/vis Spectrophotometer (Thermo Fisher Scientific, Miami, USA). Circular dichroism (CD) spectra were recorded using a Chirascan Series Spectrometer (Applied Photophysics, Surrey, UK) to analyze the secondary and tertiary structures.

Protein samples, adjusted to suitable concentrations, were analyzed for thermal stability using an Uncle multifunctional protein stability analysis system (Unchained Labs, California, USA). The samples were heated from 15°C to 95°C to assess their thermal stability across a range of concentrations.

### 2.4 SEC Analysis

A Superdex 200 10/300 GL column (GE Healthcare, UK, No. 29‐4038‐41) was calibrated with a standard gel filtration kit and utilized for SEC analyses (Figure S2). Protein samples were prepared at concentrations of 20, 50, and 100 µM. These samples were injected onto the column and separated based on molecular size at a flow rate of 0.4 mL min^−1^, maintained at 4°C.

### 2.5 Free Thiol and Protein Solubility Measurement

The molar concentration of free thiols in purified proteins was determined using a micro total mercapto assay kit (Solarbio, Beijing, China). Following the manufacturer's instructions, protein solutions were mixed with the assay reagent, and absorbance was measured using a microplate reader (BIO‐RAD Life Science, California, USA) to quantify the free thiol content.

Purified proteins were concentrated via centrifugation at 12,000 rpm for 15 min using Millipore concentrators. The relative solubility of the proteins at 37°C was then evaluated using a Nanodrop spectrophotometer (Thermo Fisher Scientific, USA). The maximum concentration achieved post‐centrifugation was recorded as the protein solubility.

### 2.6 Bioinformatics Analysis

To assess the pathogenicity of the mutation, we utilized bioinformatic tools including Mutation Taster, PolyPhen‐2, PANTHER, and SNPs&GO, all accessed through their respective online platforms. Protein hydrophobicity was evaluated using ProtScale, and three‐dimensional protein structures were modeled using the SWISS‐MODEL server. Molecular dynamics simulations were conducted with GROMACS (version 2018) employing the CHARMM36 force field to investigate protein dynamics. Additionally, schematic diagrams were created using PyMOL software for visualization and structural representation.

### 2.7 The Cataract‐Related Cell Model and Immunofluorescence Technique

HEK 293T cell lines, sourced from the American Type Culture Collection (Manassas, VA, USA), were employed to develop cellular models for protein expression [20]. These cells were cultured in DMEM basic medium (Corning, New York, USA) supplemented with 10% fetal bovine serum (FBS; AusGeneX, Gold Coast, Australia) and maintained at 37°C with 5% CO₂ in a humidified incubator. Transfection was conducted when the cell density reached 70–80%, using recombinant plasmids tagged with N‐terminal EGFP and HiEff Trans Liposomal Transfection Reagent (40802ES03, YEASEN, Shanghai, China).

For immunofluorescence analysis, cells were fixed with 4% paraformaldehyde and permeabilized with 0.4% Triton X‐100 (Sangon Biotech, Shanghai, China) for 10 min. The fixed cells were blocked with 10% FBS for 20 min, followed by staining of nuclei with DAPI and labeling of protein aggregates using a p62 antibody (1:200; Thermo Fisher Scientific, Miami, USA). Immunofluorescence‐stained cells were imaged using a confocal microscope (Zeiss, Oberkochen, Germany), and ten random fields were analyzed to quantify the percentage of aggregate‐positive cells.

### 2.8 Western Blot and SDS‒PAGE

Following the collection of transfected cells, lysis was conducted using 1% NP40 (Sangon Biotech, Shanghai, China) supplemented with protease and phosphatase inhibitors (Thermo Fisher Scientific, Miami, USA). The resulting lysate, containing total (T) proteins, was centrifuged to separate it into supernatant (S) and precipitation (P) fractions. These fractions were utilized to evaluate the relative ratio of soluble to insoluble proteins in HEK 293T cells.

Proteins were transferred onto membranes and probed with specific antibodies: a mouse monoclonal antibody (1:5000; Proteintech, USA) targeting the EGFP‐tagged fusion protein and a rabbit polyclonal antibody (1:10000; Proteintech, USA) for the mCherry‐tagged fusion protein. Membranes were imaged using the Amersham Imager 680 (Cytiva, Munich, Germany), and the intensity of the target proteins was quantified using ImageJ software (National Institutes of Health, USA). Results were normalized to the intensity of GAPDH (1:10,000; Proteintech, USA). All experiments were conducted independently in triplicate.

### 2.9 Cell Viability Assay

Cell viability was assessed using the Cell Counting Kit‐8 (Yeasen Biotechnology, China). Transfected cells were incubated with the kit's reagent at 37°C for 1 h, and the optical density (OD) values were measured using a microplate reader (BIO‐RAD Life Science, California, USA).

### 2.10 Dihydroethidium (DHE) Staining

Cell samples were prepared and incubated with a 20 mM DHE (50102ES, Yeasen Biotechnology, Shanghai, China) solution at 37°C with 5% CO₂ in a cell incubator. Following incubation, the cells were washed with PBS buffer, and ten random fluorescent images of live cells were captured using an inverted fluorescence microscope (DM i8, Leica, Germany). The fluorescence intensity of DHE was then analyzed using ImageJ software (National Institutes of Health, USA).

### 2.11 Transmission Electron Microscopy

After glow discharge treatment, protein samples were applied to copper grids (Zhongjingkeyi Technology Co., Ltd., China, BZ11033a). The samples were then stained with 3% uranyl acetate in preparation for transmission electron microscopy (TEM) analysis. Subsequently, the samples were examined, and images were captured using a Talo L120C TEM (Thermo Scientific, USA).

### 2.12 Statistical Analysis

All data were analyzed using two‐tailed Student‘’ *t*‐tests with GraphPad Prism 8, with *p* values less than 0.05 indicating statistical significance. For the imaging data, statistical results were derived from ten randomly selected fields of view, and all other experiments were repeated three times (biological replicates).

## 3 Results

### 3.1 Cataract‐Related Mutant R188C of βB2‐Crystallin Leads to Protein Aggregation

The family under analysis includes two affected individuals and one unaffected individual. The proband (II:1), indicated by a black arrow in the graphical abstract, is a 2‐year‐old boy who first exhibited lens opacity at 6 months of age. The opacity primarily impacted the posterior pole of the lens, resulting from abnormal crystallin aggregation. His father (I:2) was similarly diagnosed with bilateral posterior pole cataracts and impaired vision at an early age [21].

Targeted exome sequencing (TES) revealed a c.562C>T (p.R188C) mutation in the *βB2‐crystallin* gene in patients with congenital cataracts. Sanger sequencing validated that this mutation segregated with the disease phenotype and was not present in 100 healthy controls. Multiple sequence alignments demonstrated that the arginine (R) residue at position 188 of βB2‐crystallin is highly conserved across species, highlighting its evolutionary importance. As illustrated in the graphical abstract, these findings underscore the crucial role of the R188 region in maintaining the structural stability and hydrophobicity of *βB2‐crystallin*, which is essential for its function in vertebrates. Moreover, bioinformatics analyses classified the *βB2‐R188C* mutation as likely damaging and pathogenic (Table 1).

**TABLE 1:** Disease‐causing analysis of *CRYβB2‐R188C* mutant.

Gene	DNA alteration	Amino alteration	PolyPhen‐2	Mutation taster	PANTHER	SNPs&GO
*CRYβB2*	c. 562C>T	p.R188C	Probably damaging	Disease‐causing	Probably damaging	Disease‐causing

**FIGURE 1:**
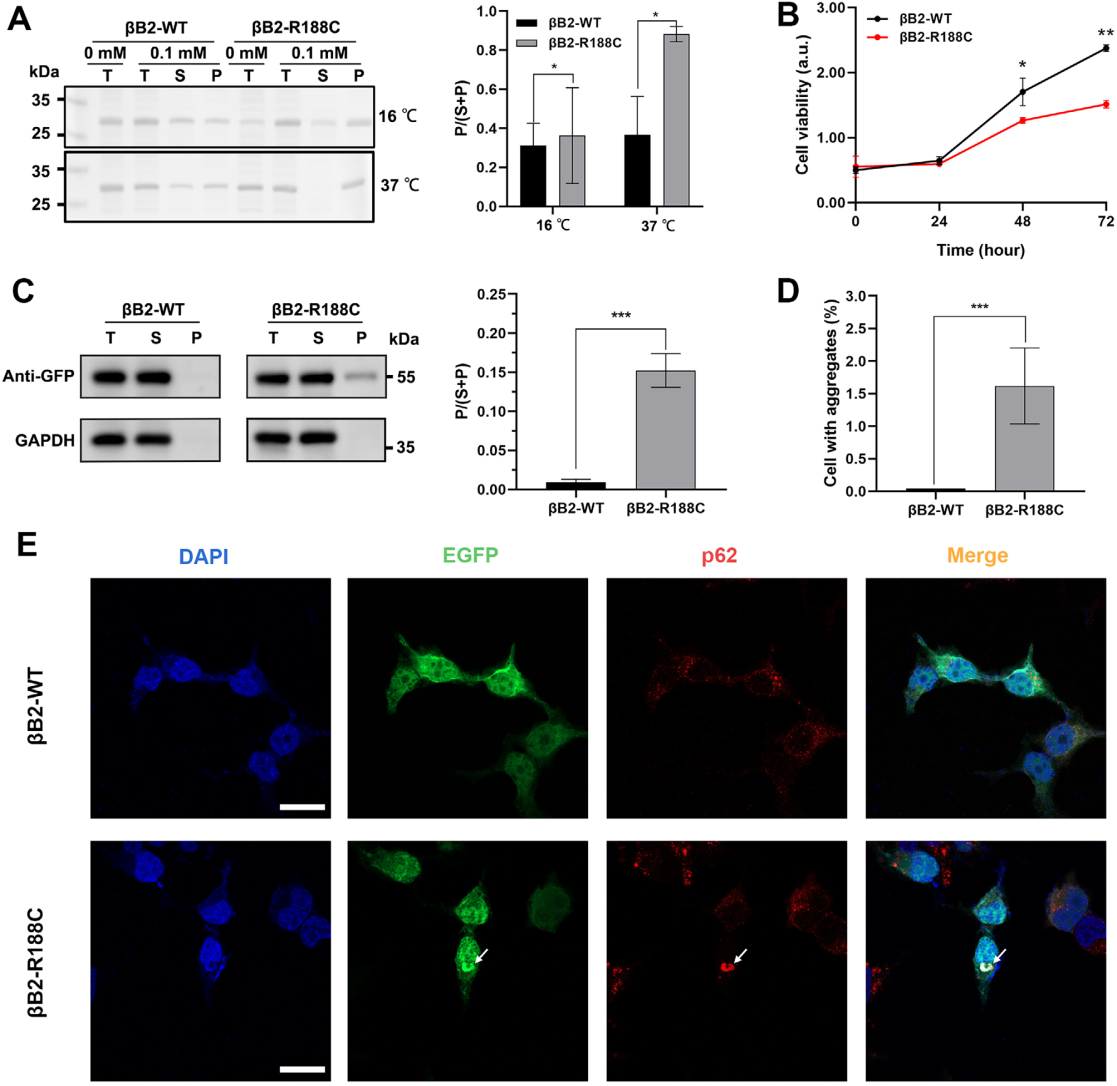
βB2‐R188C mutant tended to form aggregates and decreased cell viability. (A) Protein solubility of WT and R188C proteins expressed in Rosetta cells was analyzed via SDS‒PAGE, and determined by densitometric analysis. T: total lysate; S: supernatant; P: pellet. Different IPTG concentrations and temperatures were tested (37°C and 16°C). (B) The effect of βB2‐R188C mutant on cell viability was assessed using the CCK‐8 assay, measuring optical density (OD) at 450 nm wavelength. (C) Western blot analysis of transfected HEK 293T cells. Protein levels were determined by densitometric analysis and normalized to GAPDH. (D) Proportion of cells with aggregates. (E) Confocal images of HEK 293T cells overexpressing EGFP‐fused WT and R188C mutant proteins. Nuclei were stained with DAPI (blue). The EGFP tag was used for protein visualization, while p62 (red) marked protein aggregates. Scale bars: 10 µm. * means *p* < 0.05, ** means *p* < 0.01, and *** means *p* < 0.001.

To investigate the functional impact of this mutation, recombinant plasmids encoding the wild‐type (WT) and R188C mutant proteins were transfected into *Rosetta* cells. Under 37°C, the WT protein was evenly distributed between the supernatant and precipitate. In contrast, SDS‐PAGE analysis revealed that a substantial fraction of the R188C mutant protein accumulated in the precipitate, suggesting abnormal aggregation. Notably, when the expression temperature was reduced to 16°C, the proportion of R188C protein in the soluble fraction increased significantly (Figure 1A). This suggests that under relatively lower temperature conditions, the mutated protein is more likely to maintain a soluble, native conformation, which facilitates protein purification and subsequent analysis.

In addition to assessing protein expression in prokaryotic cells, we also evaluated expression in a eukaryotic cell system. Unlike the EGFP‐tagged wild‐type (WT) protein, the R188C mutant protein formed intracellular aggregates that colocalized with p62, a well‐established marker of protein aggregates (Figure 1D,E). Western blot analysis revealed that βB2‐WT remained largely soluble, whereas the R188C mutant protein was present in both the soluble and insoluble fractions, with the latter predominantly containing abnormal aggregates (Figure 1C). Moreover, the formation of R188C protein aggregates significantly inhibited cell proliferation, as demonstrated by cell viability assays and western blot analysis (Figure 1B; Figure S8).

### 3.2 R188C Mutation Impairs the Structure and Oligomeric Balance of βB2‐Crystallin

**FIGURE 2:**
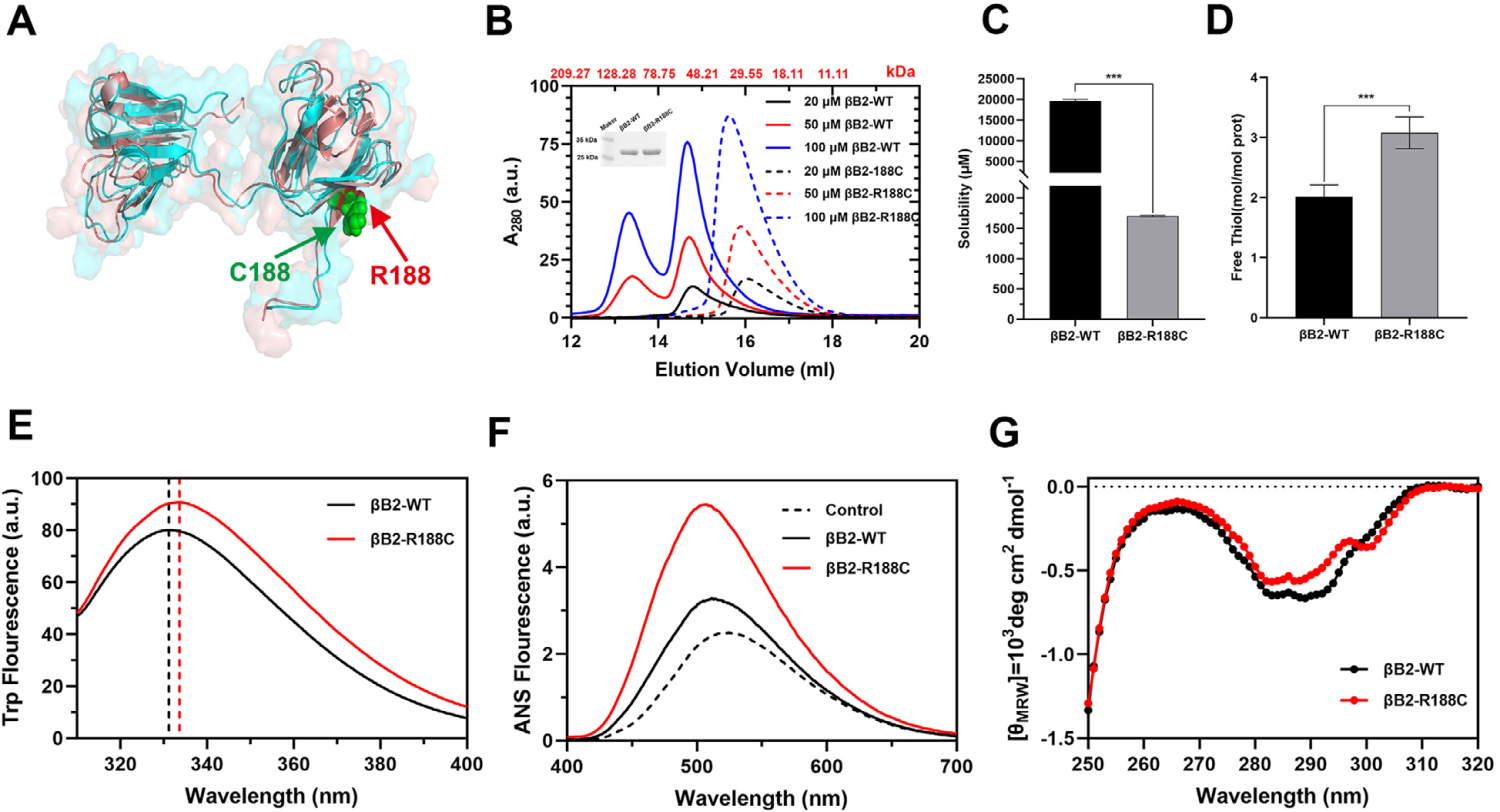
Biophysical properties of βB2‐WT and the βB2‐R188C mutant. (A) Monomer structures of βB2‐WT (cyan) and βB2‐R188C (brown). (B) SEC results for various concentrations of βB2‐WT and the βB2‐R188C mutant. The inset image shows SDS‐PAGE analysis of purified wild‐type βB2 and the R188C mutant. (C) Solubility comparison between βB2‐WT and βB2‐R188C. (D) Number of free thiols in βB2‐WT and βB2‐R188C. (E) Intrinsic Trp fluorescence spectra are excited at 295 nm. (F) Extrinsic 1‐aniline‐8‐naphthalene sulfonate fluorescence spectra excited at 380 nm. (G) Near‐UV CD spectra of βB2‐WT and βB2‐R188C at a protein concentration of 50 µM. CD spectra values are presented as the mean residue molar ellipticity [(*θ*_MRW_)]. * means *p* < 0.05, ** means *p* < 0.01, and *** means *p* < 0.001.

Biophysical analyses demonstrated that the R188C mutation, situated in the fourth Greek key motif of βB2‐crystallin, severely undermines the protein's structural stability (Figure 2A). This mutation also perturbs the oligomeric equilibrium of βB2‐crystallin. Affinity and size‐exclusion chromatography (SEC) of purified βB2‐crystallin WT and R188C mutants revealed distinct oligomerization patterns: the WT protein primarily existed as dimers and tetramers, wheras the R188C mutant predominantly formed monomers (Figure 2B). Additionally, the solubility of the R188C protein was significantly decreased to 42 mg mL^−1^ (approximately 1680 µM), compared to that of the WT protein (Figure 2C). The substitution of arginine with cysteine at position 188 introduced additional free thiols that did not participate in disulfide bond formation, as indicated by the presence of unbound sulfhydryl groups (Figure 2D).

Tryptophan fluorescence analysis (excitation at 295 nm) revealed a redshift in the emission peak from 331 nm (WT) to 334 nm (R188C), indicating alterations in the microenvironment of tryptophan residues and a shift of the hydrophobic core toward a more hydrophilic environment, resulting in a looser protein structure (Figure 2E; Figure S1). Additionally, increased extrinsic ANS fluorescence in the R188C mutant highlighted the exposure of hydrophobic side chains (Figure 2F). Differences in the near‐UV circular dichroism (CD) spectrum within the 290–310 nm range further supported increased tryptophan exposure in the mutant compared to the WT protein (Figure 2G). Collectively, these findings suggest that the R188C mutation destabilizes βB2‐crystallin, disrupts its oligomeric balance, and exposes hydrophobic regions, thereby increasing its propensity for aggregation.

### 3.3 R188C mutation Increases Susceptibility of βB2‐Crystallin to UV‐Induced Aggregation

**FIGURE 3:**
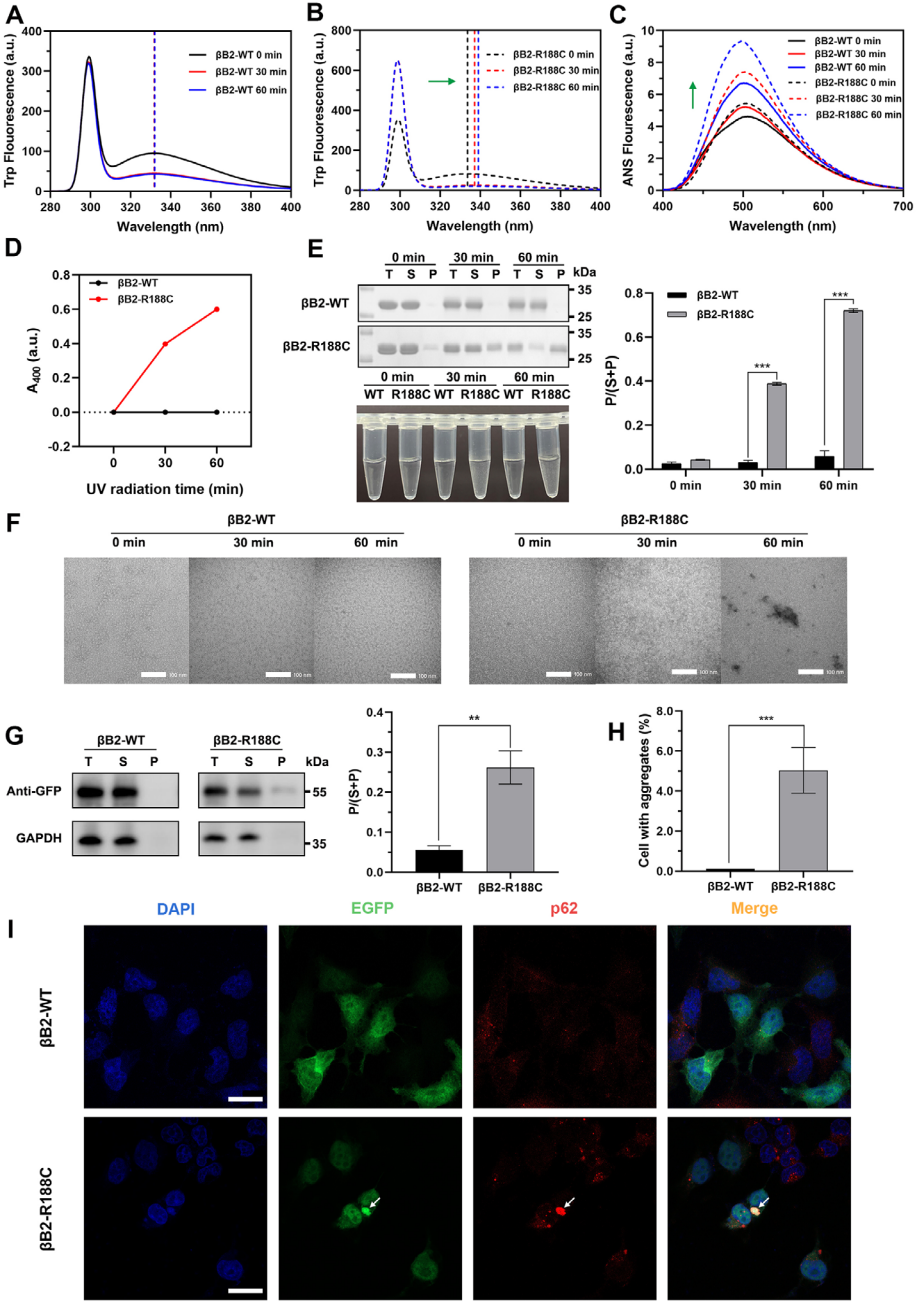
The R188C mutation increases βB2‐crystallin susceptibility to UV irradiation. (A,B) Intrinsic Trp fluorescence of βB2‐WT and βB2‐R188C after UV irradiation. (C) ANS fluorescence of βB2‐WT and βB2‐R188C after UV irradiation. (D) Turbidity at 400 nm of βB2‐WT and βB2‐R188C after UV irradiation. (E) SDS‐PAGE with densitometric analysis of βB2‐WT and βB2‐R188C after UV irradiation. (F) Transmission electron microscopy image of βB2‐WT and βB2‐R188C under varying durations of UV treatment. (G) Western blot analysis of transfected HEK 293T cells after 30 min of UV irradiation. Relative protein levels (P / (S + P)) were determined by densitometric analysis and normalized to GAPDH. (H) Proportion of cells with aggregates after 30 min of UV irradiation. (I) Confocal images of HEK 293T cells overexpressing EGFP‐fused WT and R188C mutant proteins after 30 min of UV irradiation. Nuclei were stained with DAPI (blue). Protein aggregates are recognized by the p62 marker protein (red). Scale bars: 10 µm. n = 3; * means *p* < 0.05, ** means *p* < 0.01, and *** means *p* < 0.001.

In addition to studying the effects of the R188C mutation under physiological conditions, we also assessed its impact under environmental stressors commonly encountered by the eye, including UV radiation, oxidative stress, and heat stress. To further investigate, βB2‐WT and R188C constructs were subjected to UV radiation at both protein and cellular levels. The Trp fluorescence of βB2‐WT remained stable after 30 and 60 min of UV exposure (Figure 3A), whereas the R188C mutant exhibited progressive redshifts in its emission peak, shifting from 334 to 337 nm and further to 339 nm, reflecting the destabilization of βB2‐crystallin under UV irradiation (Figure 3B). Similar trends were observed in extrinsic ANS fluorescence, where increased hydrophobic surface exposure was evident for the R188C mutant (Figure 3C). Moreover, the R188C mutant exhibited a significantly higher propensity for aggregation, forming larger and denser protein complexes compared to βB2‐WT under UV radiation (Figure 3D–F). In the cellular model, overexpression of the R188C mutant resulted in a marked increase in intracellular p62‐positive aggregates following UV treatment, correlating with a significantly higher proportion of insoluble precipitated proteins in the R188C group under UV irradiation (Figure 3G–I). These results demonstrate that the R188C mutation compromises the structural stability of βB2‐crystallin, rendering it more susceptible to forming abnormal protein aggregates when exposed to UV stress.

### 3.4 The R188C Mutation Sensitizes βB2‐Crystallin to Oxidative Stress

**FIGURE 4:**
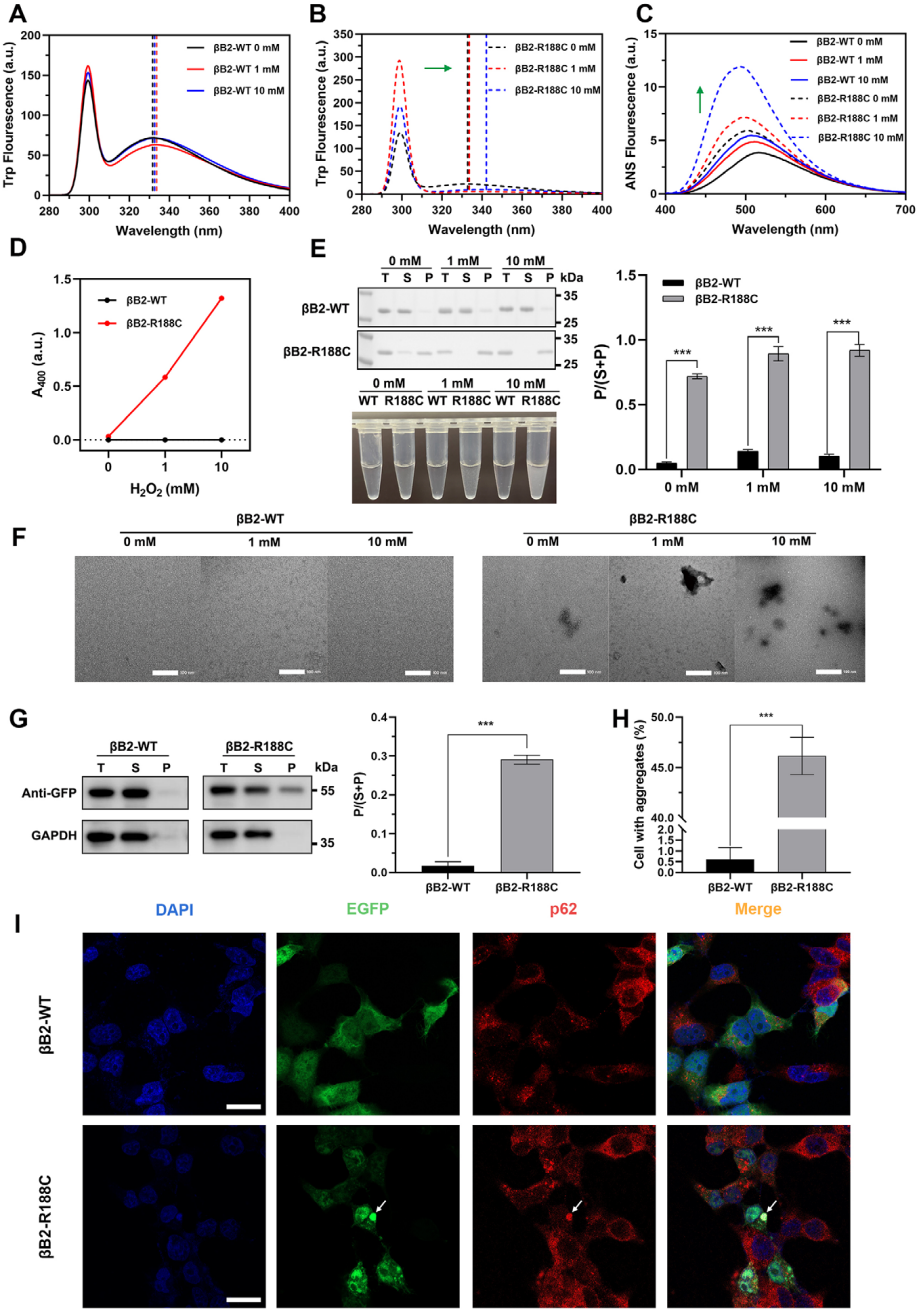
The R188C mutation increases βB2‐crystallin sensitivity to oxidative stress. (A,B) Intrinsic Trp fluorescence of βB2‐WT and βB2‐R188C under different levels of oxidative stress. (C) ANS fluorescence of βB2‐WT and βB2‐R188C under different levels of oxidative stress. (D) Turbidity at 400 nm of βB2‐WT and βB2‐R188C under different levels of oxidative stress. (E) SDS‒PAGE with densitometric analysis of βB2‐WT and βB2‐R188C under different levels of oxidative stress. (F) Transmission electron microscopy image of βB2‐WT and βB2‐R188C treated with different concentrations of H_2_O_2_. (G) Western blot analysis of transfected HEK 293T cells after 1 h of oxidation. Relative protein levels (P / (S + P)) were determined by densitometric analysis and normalized to GAPDH. (H) Proportion of cells with aggregates after 1 h of 1 mM H_2_O_2_ treatment. (I) Confocal images of HEK 293T cells overexpressing EGFP‐fused WT and R188C mutant proteins after 1 h of 1 mM H_2_O_2_ treatment. Nuclei were stained with DAPI (blue). Protein aggregates recognized by the p62 marker protein (red). Scale bars: 10 µm. n = 3, * means *p* < 0.05, ** means *p* < 0.01, and *** means *p* < 0.001.

Expanding on our findings that the R188C mutation sensitizes βB2‐crystallin to UV‐induced aggregation, we explored its response to oxidative stress, another key environmental factor implicated in cataract formation. After H_2_O_2_ treatment, HEK 293T cells produced a large amount of reactive oxygen species, which showed red fluorescence under a fluorescence microscope after DHE staining (Figure S7). Our investigation revealed that oxidative stress significantly exacerbated the formation of abnormal protein aggregates in βB2‐crystallin. Treatment with varying concentrations of H_2_O_2_ showed that βB2‐R188C exhibited markedly increased hydrophobicity and pronounced alterations in the tryptophan microenvironment compared to βB2‐WT, as evidenced by differences in intrinsic and extrinsic fluorescence (Figure 4A–C). Furthermore, βB2‐R188C demonstrated a higher tendency to develop turbidity and form large aggregates upon exposure to H_2_O_2_ (Figure 4D,E). Additionally, TEM revealed extensive protein aggregation in βB2‐R188C following incubation with 1 and 10 mM H_2_O_2_ at 37°C for 12 h (Figure 4F). These findings were consistent across both purified protein samples and HEK 293T cells, where R188C‐transfected cells displayed a significantly higher propensity to form intracellular p62‐positive aggregates under 1 mM H_2_O_2_ treatment, as confirmed by confocal imaging and aggregate quantification (Figure 4G–I).

### 3.5 R188C Mutant Promotes Protein Aggregation Under Heat Stress

**FIGURE 5:**
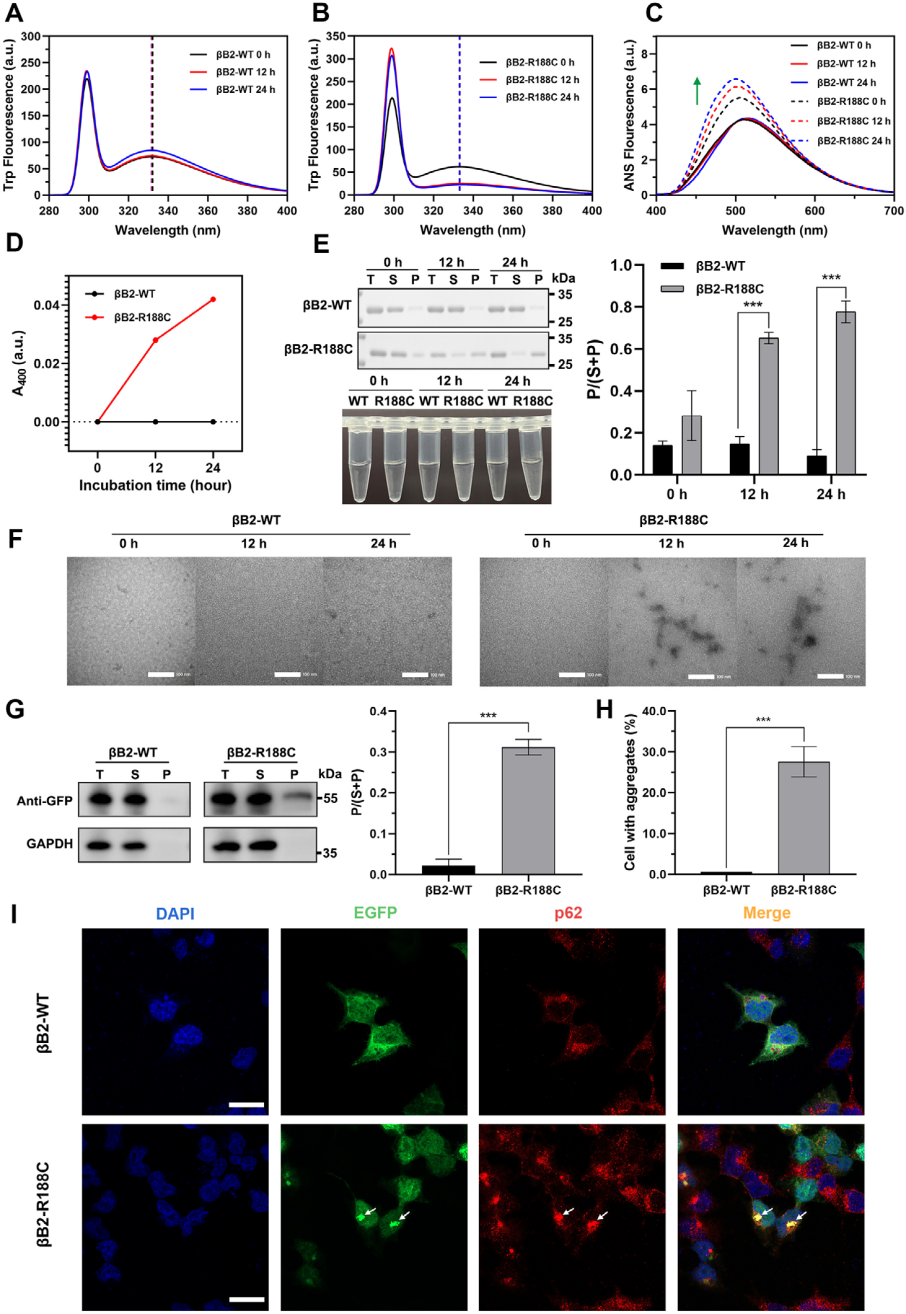
The R188C mutation tends to be more unstable under heat stress. (A,B) Intrinsic Trp fluorescence of βB2‐WT and βB2‐R188C after 12 and 24 h durations of 37°C incubation. (C) ANS fluorescence of βB2‐WT and βB2‐R188C after 12 and 24 h durations of 37°C incubation. (D) Turbidity at 400 nm of βB2‐WT and βB2‐R188C after 12 and 24 h durations of 37°C incubation. (E) SDS‒PAGE and densitometric analysis of βB2‐WT and βB2‐R188C after 12 and 24 h durations of 37°C incubation. (F) Transmission electron microscopy images of βB2‐WT and βB2‐R188C after 12 and 24 h durations of 37°C incubation. (G) Western blot analysis of transfected HEK 293T cells after 1 h of 42°C heat shock. Relative protein levels (P / (S + P)) were determined by densitometric analysis and normalized to GAPDH. (H) Proportion of cells with aggregates after 1 h of 42°C heat shock. (I) Confocal images of HEK 293T cells overexpressing EGFP‐fused WT and R188C mutant proteins after 1 h of 42°C heat shock. Nuclei were stained with DAPI (blue). Protein aggregates detected by p62 marker protein (red). Scale bars: 10 µm. n = 3; * means *p* < 0.05, ** means *p* < 0.01, and *** means *p* < 0.001.

Extending our investigation of the R188C mutation's sensitivity to environmental stressors, we examined its behavior under heat stress, which is another significant factor contributing to protein aggregation. Our findings indicate that βB2‐R188C is particularly sensitive to temperature changes, as evidenced by its tendency to aggregate and precipitate even at physiological temperature. To elucidate this sensitivity, we analyzed the Trp and ANS fluorescence of purified βB2‐WT and βB2‐R188C proteins after incubation at 37°C. Although no significant shift in the peak position of Trp fluorescence was observed for either protein (Figure 5A,B), a marked increase in ANS fluorescence was detected in R188C after 12 h of incubation, suggesting enhanced exposure of hydrophobic regions (Figure 5C). Subsequent turbidity assays, SDSPAGE analysis, and TEM imaging corroborated these observations, revealing that R188C is prone to forming heterogeneous aggregates under prolonged incubation (Figure 5D–F). Additionally, we assessed the stability of the R188C mutant in cell models under heat stress. Consistent with our above findings, the R188C mutant displayed a significantly higher precipitate proportion than βB2‐WT. Furthermore, the colocalization of p62‐positive protein aggregates was more prominent in R188C‐transfected cells than in WT‐transfected cells under heat stress (Figure 5G–I).

### 3.6 The R188C Mutant Compromises Thermal Stability

**FIGURE 6:**
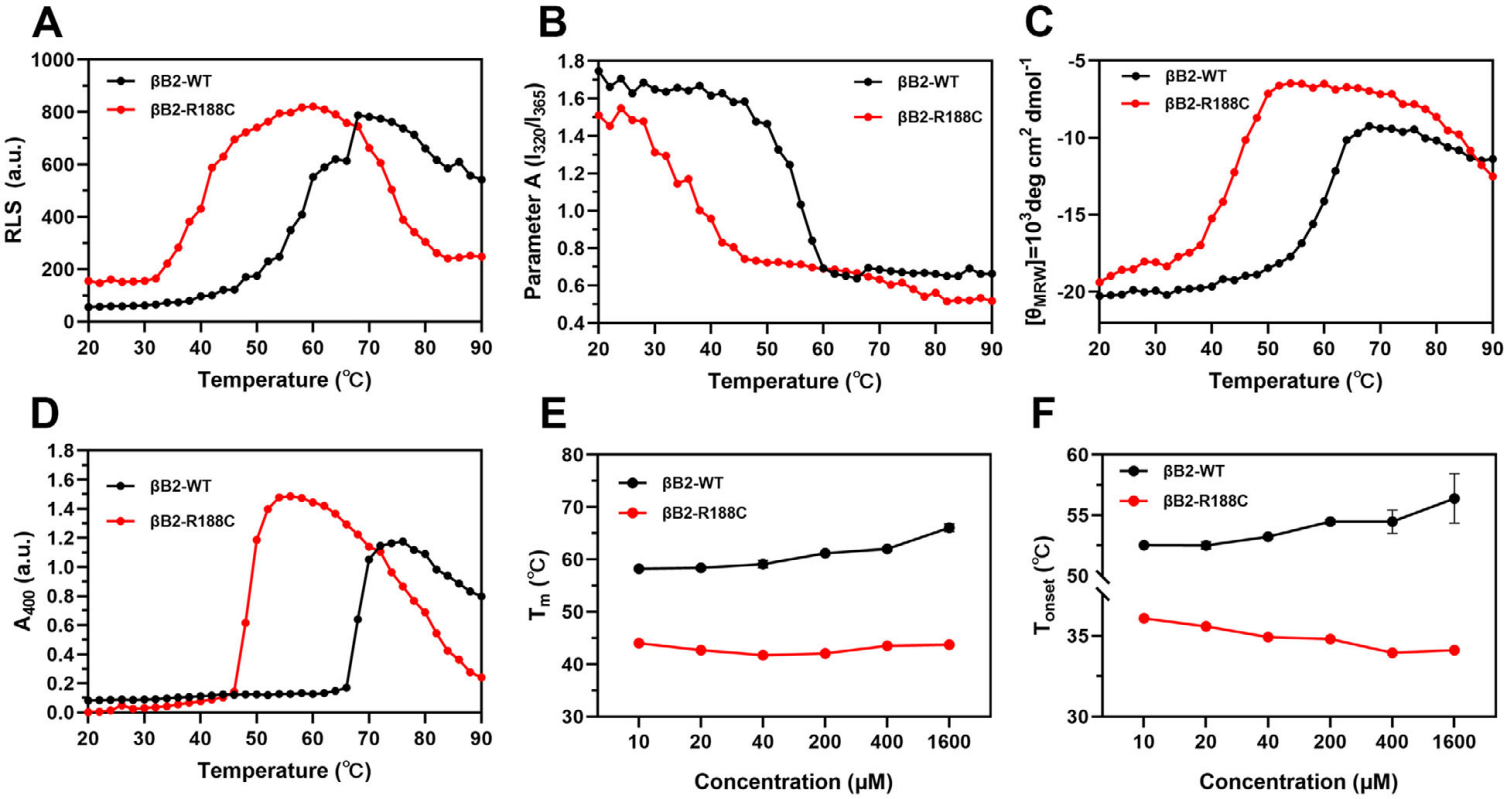
The thermal stability of βB2‐crystallin is significantly impaired by the R188C mutation. (A) Resonance light scattering (RLS) spectra of βB2‐WT and βB2‐R188C during a temperature experiment. (B) Intrinsic Trp fluorescence at 295 nm of βB2‐WT and βB2‐R188C during a temperature experiment. (C) Molar ellipticity of far‐UV CD at 222 nm of βB2‐WT and βB2‐R188C during a temperature experiment. (D) Turbidity at 400 nm of βB2‐WT and βB2‐R188C. (E) Denaturation midpoint temperature (*T*_m_) of βB2‐WT and βB2‐R188C. (F) The denaturation onset temperature of βB2‐WT and βB2‐R188C.

To further investigate the effects of the R188C mutation under heat stress, we employed resonance light scattering (RLS), absorbance at 400 nm (*A*_400_), parameter A (*I*_320_/*I*_365_), and far‐UV CD spectroscopy to assess conformational and structural changes during thermal denaturation. RLS directly reflects changes in soluble protein aggregates and particle size. During the heating process, βB2‐R188C formed soluble aggregates at 32°C and insoluble aggregates at 46°C, much earlier than βB2‐WT (Figure 6A,D). Additionally, parameter A of βB2‐R188C exhibited a sharp decline at approximately 33°C, significantly lower than the corresponding value for βB2‐WT, indicating that the R188C mutant protein adopted a more relaxed structure and exposes tryptophan groups to the solvent phase at a lower temperature (Figure 6B). Furthermore, the secondary structure of βB2‐R188C was more vulnerable to thermal changes, with structural disruption beginning at 32°C, compared to the higher thermal tolerance of βB2‐WT (Figure 6C).

Thermal stability indicators, including melting temperature (*T*_m_) and onset temperature (*T*_onset_), further highlighted the compromised stability of βB2‐R188C. For βB2‐WT, *T*_m_ increased with protein concentration, ranging from 56°C to 66°C, and *T*_onset_ rose from 52.5°C to 57°C. In contrast, βB2‐R188C exhibited a progressive decrease in *T*_m_ with increasing protein concentration, while *T*_onset_ declined from 36°C to 33°C, underscoring its heightened susceptibility to thermal denaturation (Figure 6E,F). Beyond the results mentioned above, complementary experimental data including maximum emission wavelength of intrinsic Trp fluorescence (*E*_max_), barycentric mean (BCM), and static light scattering (SLS) further confirmed the compromised thermostability of the protein caused by the R188C mutation (Figure S5). Overall, these findings demonstrated that the R188C mutation severely impaired the spatial and structural stability of βB2‐crystallin, leading to aggregate formation at physiological temperature and heightened sensitivity to environmental stresses. The accumulation of these protein aggregates compromised lens transparency, ultimately contributing to cataract formation.

### 3.7 The R188C Variant Broke the Oligomeric Equilibrium of βB2‐Crystallin and Influenced Inter‐Subunit Interaction

**FIGURE 7:**
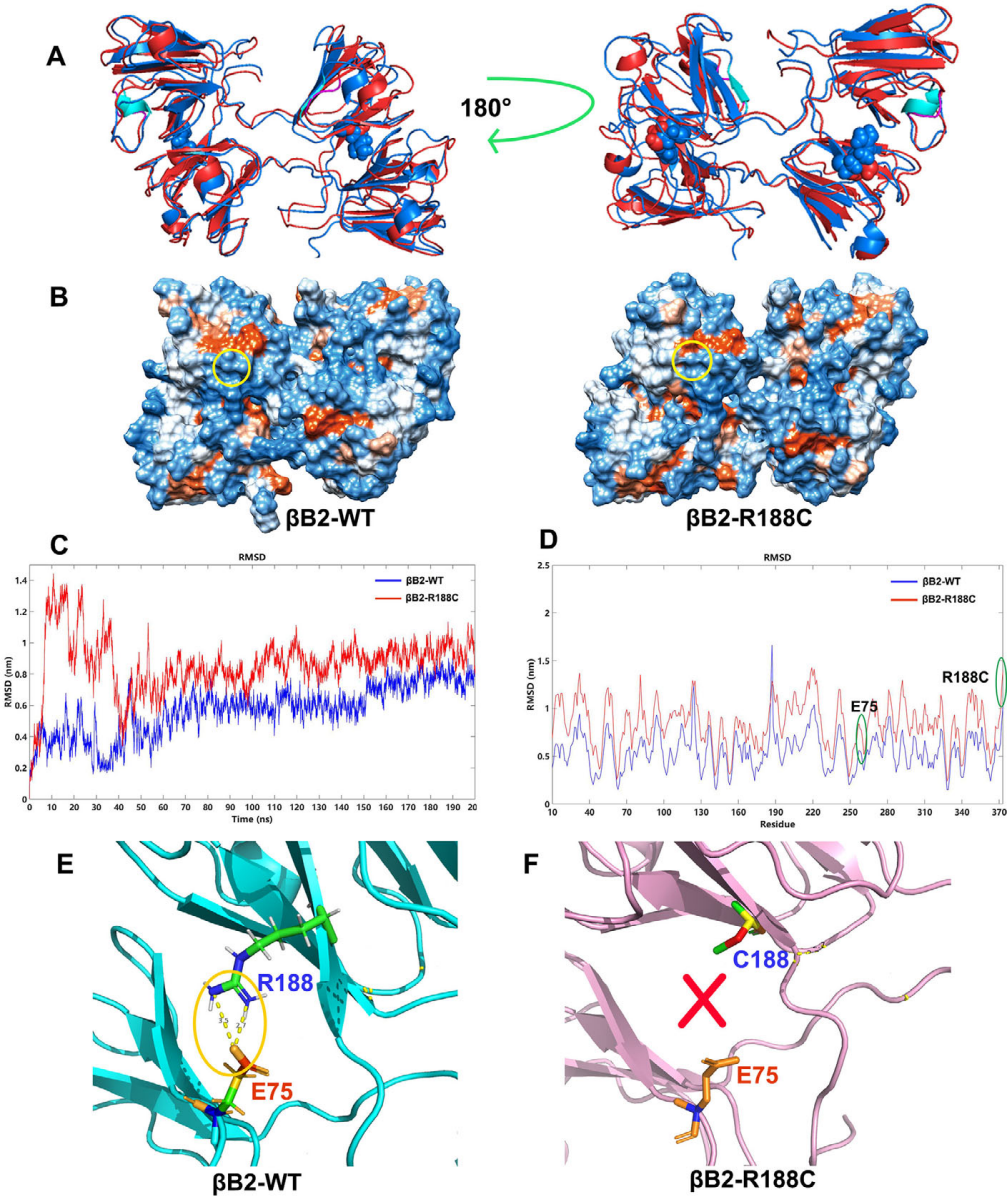
R188C mutation damaged dismeric structure of βB2‐crystallin. (A) Comparison of the well‐executed simulated dimeric structures between WT (blue) and R188C (red). The α‐helix and β‐sheet (cyan) transform into loops (magenta) in the R188C mutant, with the mutation site represented by a sphere. (B) Increased hydrophobicity around the mutation site is evident. Hydrophilic and hydrophobic residues are marked in blue and orange, respectively. The mutant site is encircled by a yellow dotted line. (C) Time‐course root‐mean‐square deviation (RMSD) from the simulated structure. (D) RMSD comparison between wild‐type and R188C proteins. The positions of R188C and E75 are highlighted with green circles. (E) R188 forms intersubunit ion pairs with E75 in βB2‐WT. (F) The ion pair between R188 and E75 was destroyed by the R188C mutation.

Molecular dynamics simulations of the WT and R188C variants revealed that the R188C mutation significantly altered the secondary structures of βB2‐crystallin. Specifically, in chain A, the α‐helix (82–85 W) and β‐sheet (169G‐171Y) were transformed into loops for the R188C variant (Figure 7A). This observation is consistent with the findings from the above‐mentioned fluorescence experiments, indicating increased exposure of hydrophobic residues in the R188C mutant (Figure 7B).

The root‐mean‐square deviation (RMSD) of the R188C mutant significantly increased initially, indicating that the mutation destablized the simulation system and disrupted stable dimer formation (Figure 7C). Interestingly, the surrounding residues of E75 and C188 in βB2‐R188C exhibited dramatically higher average residue RMSD compared to βB2‐WT (Figure 7D). Additionally, our research indicated that the mutation interfered with the interaction between R188 and E75 within the dimer of βB2‐crystallin, potentially leading to disordered interaction in this conserved domain (Figure 7E,F).

**FIGURE 8:**
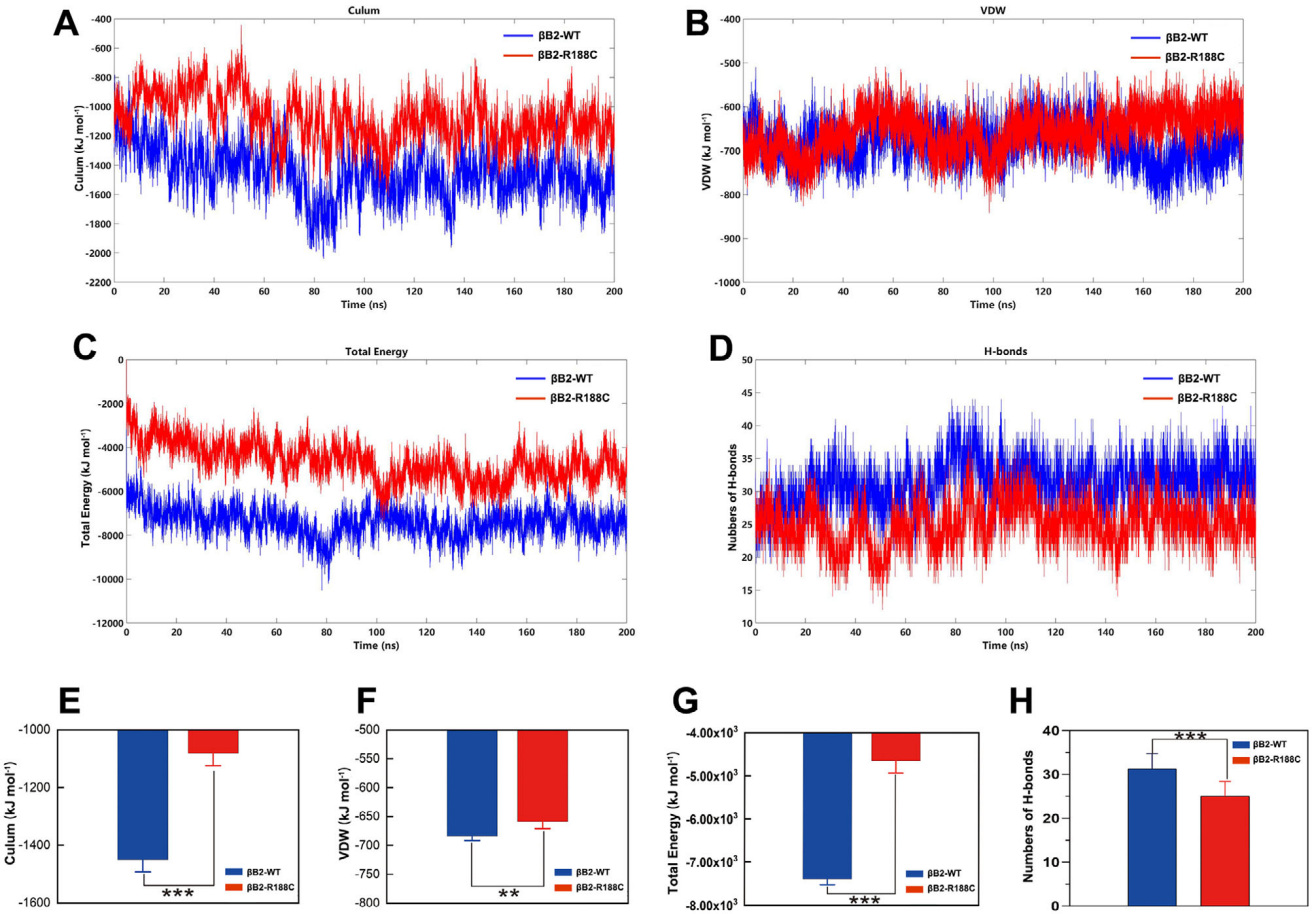
Disruption of the intersubunit ion pairs between R188C and E75 by the R188C mutation. (A–D) Time‐course analysis of Coulomb force, van der Waals force, total subunit binding energies, and the number of H‐bonds in WT and R188C. (E–H) Comparative analysis of the results from A–D. n = 3, * means *p* < 0.05, ** means *p* < 0.01, and *** means *p* < 0.001.

Both intermolecular and intramolecular forces are critical determinants of protein behavior. Our study demonstrated that the binding energy,‐comprising Coulomb forces, van der Waals forces, and total subunit binding energies between the monomers of the mutant protein were significantly higher than that of the wild‐type protein (Figure 8A–C,E–G). This increased binding energy in βB2‐R188C suggests a reduced ability of the mutant protein to form stable dimers. Furthermore, hydrogen bonds, which are essential for maintaining structural stability, were markedly reduced in βB2‐R188C compared to the wild type (Figure 8D,H). Collectively, these results demonstrate that the R188C mutation disrupts spatial interactions between βB2‐crystallin subunits, thereby weakening structural stability and promoting the formation of abnormal protein aggregates.

### 3.8 αB‐Crystallin and Lanosterol Targeting Mitigates Protein Aggregation

**FIGURE 9:**
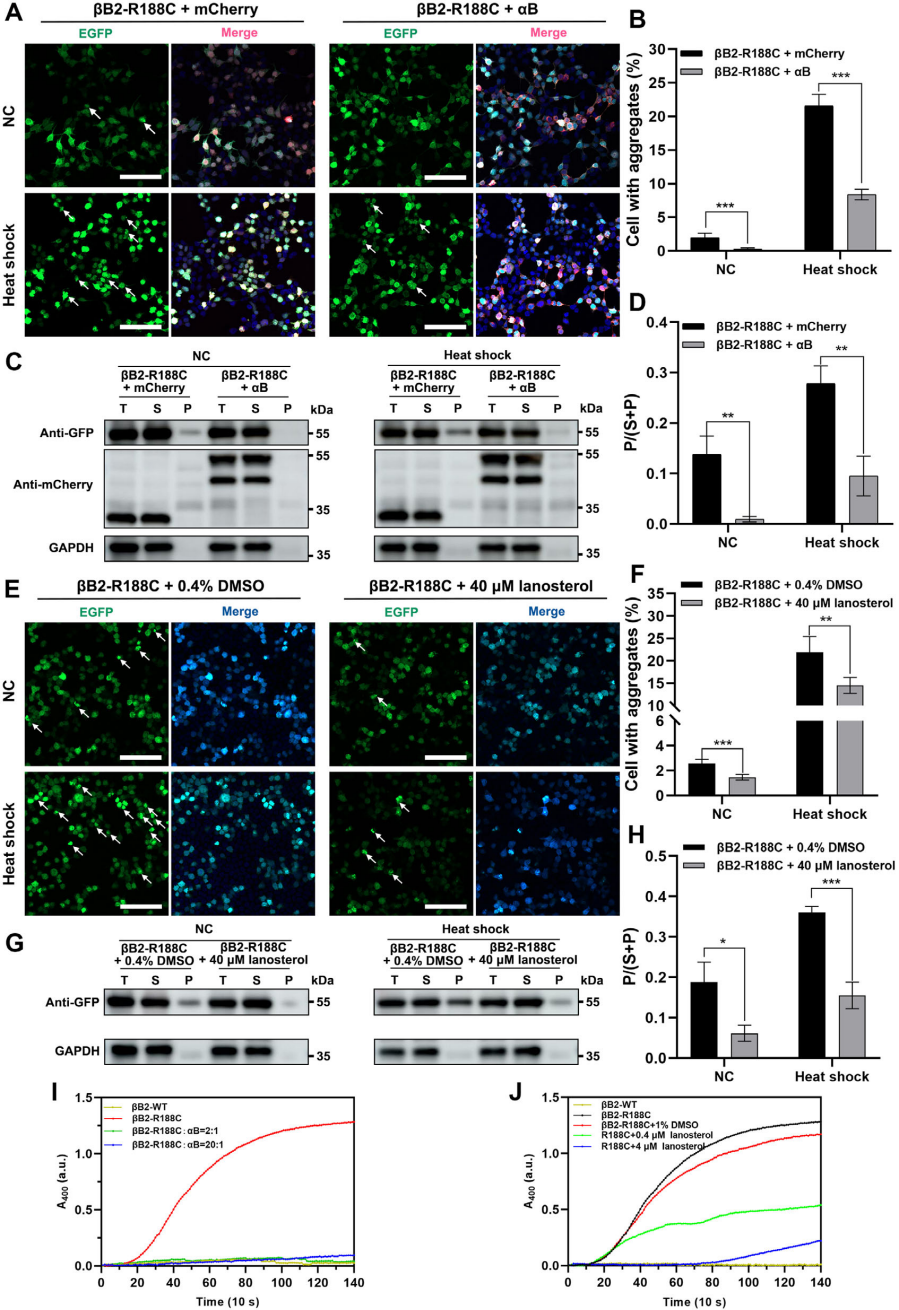
The thermal denaturation of the mutant protein R188C can be partially alleviated by adding lanosterol and αB‐crystallin. (A) HEK 293T cells were co‐transfected with αB‐crystallin and βB2‐R188C plasmids, and then cultured in normal condition or 1 h of 42°°C heat shock. (B) Aggregation proportion, (C) western blot analysis, and (D) densitometric analysis of the two groups were shown. (E) HEK 293T cells were transfected with βB2‐R188C plasmids, and treated with 40 µM lanosterol, and then cultured in normal condition or 1 h of 42°°C heat shock. (F) Aggregation proportion, (G) western blot analysis, and (H) densitometric analysis of the two groups were shown. (I) Turbidity at 400 nm of βB2‐WT and βB2‐R188C under 45°C high‐temperature stress after mixing with different concentrations of αB‐crystallin. (J) Turbidity at 400 nm of βB2‐WT and βB2‐R188C under 45°C high‐temperature stress after the administration of different concentrations of lanosterol. Scale bars: 100 µm. n = 3, * means *p* < 0.05, ** means *p* < 0.01, and *** means *p* < 0.001.

αB‐crystallin, a small heat shock protein, is known to protect β‐crystallin from thermal denaturation [16, 22]. In our study, co‐transfection of αB‐crystallin with the βB2‐R188C mutant in cells significantly reduced the formation of intracellular protein aggregates under both normal culture conditions and heat stress (Figure 9A–D,I). Lanosterol showed positive influence on the long‐term cell viability of R188C‐transfected HEK 293T cell, which may also alleviate the formation of abnormal protein aggregates (Figures 1B; Figure S9) [23]. Numerous studies have highlighted lanosterol's role in preventing lens protein aggregation [24]. Consistent with these findings, treatment with 40 µM lanosterol effectively reduced aggregate formation in cells expressing the βB2‐R188C mutant, further supporting its potential as a therapeutic agent (Figure 9E–H,J).

## 4 Discussion

Congenital cataracts are multifactorial diseases influenced by both genetic and environmental factors, with crystallin mutations contributing to approximately 45% of cases [5, 25, 26]. Among the various crystallins, βB2‐crystallin is essential for maintaining lens transparency and stability [27, 28, 29]. To better understand its pathogenic mechanism, our study focused on the R188C mutation in βB2‐crystallin, revealing its impact on structural integrity and susceptibility to stress‐induced aggregation.

Specifically, the R188C mutation severely compromises the structural and thermal stability of βB2‐crystallin. Biophysical analyses revealed perturbed oligomeric equilibrium and increased exposure of hydrophobic regions, both of which correlate with a heightened propensity for aggregation under physiological and stress conditions. This finding underscores the importance of conserved Greek‐key motifs, which are critical for the stability and function of βB2‐crystallin and for preventing congenital cataract formation [28, 30, 31]. Mutations such as βB2‐G149V and βB1‐L116P, located in Greek‐key motifs 4 and 2, respectively, have been shown to severely impair the thermodynamic stability of the mutated proteins [32, 33]. Similarly, our results show that the R188 residue, positioned in Greek‐key motif 4, exhibits increased hydrophobicity, partially explaining its enhanced aggregation tendency compared to the WT protein.

Moreover, the dynamic oligomerization of β‐crystallins is vital for maintaining their stability and solubility. In particular, the terminal strand of Greek‐key motif 4 in βB2‐crystallin is essential for preserving the balance of oligomeric states and ensuring the stability of monomer interactions [34, 35]. Notably, protein concentration significantly influences protein‐protein interactions, with higher concentrations promoting oligomer formation and exhibiting concentration‐dependent oligomeric equilibrium [36, 37]. SEC analysis revealed that the βB2‐R188C mutation disrupted this equilibrium, favoring a monomeric structure. In contrast, βB2‐WT maintained a concentration‐dependent balance of dimers and tetramers, consistent with previous findings on βB2‐crystallin oligomerization dynamics [28, 38, 39].

The formation of oligomeric βB2‐crystallin is associated with specific salt bridges and inter‐subunit ion pairs, such as V74‐R187, R97‐E166, and E75‐R188, as well as two hydrophobic residue pairs, V59‐L164 and V72‐V151 [34, 40, 41]. Our findings further reveal that mutation at R188, such as R188H, partially disrupt these interactions, impairing dimer and tetramer stability. The guanidinium group (─NH₄⁺) of arginine carries a positive charge, while the carboxyl group (─COO⁻) of glutamate carries a negative charge. Under physiological conditions, they can form a stable salt bridge through electrostatic interactions. This salt bridge plays an important stabilizing role in the three‐dimensional structure of proteins. However, the R188C mutation prevents the formation of a salt bridge between E75 and R188 [42, 43]. This disruption weakens key electrostatic forces, which play a dominant role in maintaining oligomeric equilibrium, despite the involvement of weaker inter‐protein forces [37, 44]. Molecular dynamic simulations confirmed that the R188C mutation destabilizes critical intersubunit ionic interactions, particularly between R188 and E75, leading to impaired dimer formation and increased protein aggregation. Together, these findings underscore the importance of conserved Greek‐key motifs and oligomeric balance in maintaining βB2‐crystallin stability and preventing cataractogenesis.

Congenital cataracts refer to the lens opacification that exists before birth or develops gradually after birth, caused by genetic inheritance or formed during the development. In some clinical cases where the severity of cataracts continues to progress after birth, the role of these stress conditions becomes particularly critical [45]. This is because mutations reduce the tolerance of lens proteins to various stress conditions. As a result, even under normal conditions, the disease progression is accelerated. In our study, environmental stressors, including UV radiation, oxidative stress, and heating, significantly enhanced the aggregation propensity of the R188C mutant (Figure S3). This aligns with previous studies linking oxidative damage and UV exposure to cataract formation [46, 47, 48, 49]. As the temperature increases, the diffusion speed and collision probability of protein molecules increase, which are the main reasons for the accelerated formation of protein aggregates [50, 51]. In this study, the R188C mutation dramatically impaired the thermodynamic stability of βB2‐crystallin, resulting in a propensity of aggregation even under normal physiological conditions in both prokaryotic and eukaryotic cellular systems. These insights are in alignment with a recent study that highlights that Cys residues are particularly susceptible to aggregation due to modifications induced by oxidative stress [52]. Furthermore, the newest paper in Cell highlights that Cys residues promote inter‐protein disulfide bonding, thus reducing protein mobility, a phenomenon termed ‘proteolethargy’. This condition is identified as a common feature in many chronic diseases [53]. Moreover, at lower guanidine concentrations, the inner structure of the R188C protein became looser than that of the wild‐type protein, which means that the R188C protein requires less energy to break its tertiary structure, leading to denaturation and aggregation (Figure S4).

Encouragingly, our research demonstrated that both lanosterol and αB‐crystallin effectively mitigate the detrimental effects of the R188C mutation. Lanosterol, a key intermediate in the cholesterol biosynthesis pathway, has been demonstrated to enhance proteasomal proteolysis and stabilize proteins. This action facilitates the clearance of misfolded proteins and mitigates their cytotoxic effects [54, 55]. What is more, a paper published in *Nature* also demonstrated that lanosterol could alleviate the severity of cataracts in both cellular and animal models [24]. αB‐crystallin, a small heat shock protein with molecular chaperone activity, can effectively inhibit the aggregation of misfolded proteins [16, 56]. Previous studies have identified that the N‐terminal domain of αB‐crystallin is essential for modulating its chaperone function [57]. Moreover, αB‐crystallin can mitigate protein aggregation within cells by modulating the microenvironment of the endoplasmic reticulum [58]. Some traditional Chinese medicines have also been found to be useful in the treatment of cataracts. For example, resveratrol and curcumin can exert antioxidant effects and delay the formation of cataracts [59]. These findings suggest that targeting aggregate formation and enhancing chaperone activity holds promise for therapeutic interventions in cataract prevention and treatment.

Despite these advances, the study has limitations. The cell models in this study were built by transient transfection reagent, making it difficult to observe long‐term protein aggregation and cellular function changes. However, we designed experiments to culture cell models for 48 and 72 hours to explore the long‐term changes for mutant proteins and cells as much as possible (Figure 1B; Figures S6 and S8–S9). Next, in vivo models were not utilized to confirm the pathological role of the R188C mutation or the therapeutic efficacy of lanosterol and αB‐crystallin. Additionally, the use of HEK 293T cells instead of lens‐derived cell lines may limit the direct translation of findings to clinical applications. Due to the differences in growth and protein expression characteristics between 293T cells and lens epithelial cells, there are limitations in exploring cellular pathways and cell metabolism. Appropriate congenital cataracts organoid models in vitro and animal models in vivo should be used for advanced research in molecular mechanism and clinical translation [60, 61, 62]. Future research should address these gaps and explore long‐term therapeutic effects in animal models.

In summary, the R188C mutation severely undermines the structural and thermal stability of βB2‐crystallin, promoting abnormal protein aggregation under stress conditions and contributing to cataractogenesis. Targeted interventions, including lanosterol and αB‐crystallin, offer potential therapeutic strategies to mitigate these effects.

## Conflicts of Interest

The authors declare no conflicts of interest.

## Author contributions

Lidan Hu, Xiangjun Chen, Yibo Yu, and Ke Yao conceived, designed, and supervised the research. Silong Chen, Lidan Hu, Ying Zhang, Jiarui Guo, Chengpeng Wu, Wu Wei, Xiaoyu Cheng, Chenqi Luo, Jingjie Xu, and Hang Song performed the experiments. Xiangjun Chen, Yibo Yu, Jing Guo, Yip Chee Chew, Ke Yao analyzed the acquired data. Xiangjun Chen, Silong Chen, and Lidan Hu wrote this manuscript. All the authors have read and approved the final manuscript.

## Supporting information



Supporting Information

## Data Availability

All data needed to support the findings of this study are present in the paper and/or in the Supporting Information. Additional data related to this paper are available from the corresponding authors.
