# Supplementary material for: Cataract‐Causing Mutant R188C of βB2 Crystallin With Low Structural Stability is Sensitive to Environmental Stresses and Prone to Aggregates Formation
Source: Exploration (Beijing). 2025 Apr 1;5(3):20240192. doi: 10.1002/EXP.20240192 (PMC12199437; doi:10.1002/EXP.20240192)
Supplement: Supplementary file 1 — Supporting Information [file EXP2-5-20240192-s001.docx]

Supporting Information

Cataract-causing mutant R188C of βB2 crystallin with low structural stability is sensitive to environmental stresses and prone to aggregates formation

Yibo Yu ^1#^, Silong Chen ^1#^, Ying Zhang ^1, 2#^, Hang Song ^3#^, Jiarui Guo ^1#^, Chengpeng Wu ^1, 2^, Wei Wu ^1^, Jingjie Xu ^1^, Xiaoyu Cheng ^1^, Chenqi Luo ^1^, Jing Guo ^4^, Yip Chee Chew ^5^, Ke Yao ^1^, Xiangjun Chen ^1, 2*^, Lidan Hu ^6*^

1. Zhejiang University, Eye Center of Second Affiliated Hospital, School of Medicine, Hangzhou, China, 310009.

2. Institute of Translational Medicine, Zhejiang University School of Medicine, 268 Kaixuan Road, Hangzhou, China, 310020.

3. Department of Ophthalmology, Peking Union Medical College Hospital, Chinese Academy of Medical Sciences and Peking Union Medical College, Beijing, China, 100730.

4. Centre for Computational Biology (CCB), Duke-NUS Medical School, 8 College Road, Singapore, 169857.

5. Ophthalmology & Visual Sciences Department, Khoo Teck Puat Hospital, 90 Yishun Central, Singapore, 768828.

6. The Children’s Hospital, Zhejiang University School of Medicine, National Clinical Research Center for Child Health, Hangzhou, Zhejiang Province, China, 310052.

# These authors contributed equally to this work.

**Running head:** The mechanism of βB2-R188C causing cataracts.

*** Correspondence:**

**Lidan Hu,**

The Children’s Hospital, Zhejiang University School of Medicine, National Clinical Research Center for Child Health, 3333 Binsheng Road, Hangzhou 310052, Zhejiang Province, China. E-mail address: hulidan@zju.edu.cn

**Xiangjun Chen,**

Eye Center of the Second Affiliated Hospital, School of Medicine, Zhejiang University, 88 Jiefang Road, Hangzhou 310009, Zhejiang Province, China. Tel/Fax: +8657186971812; E-mail address: <chenxiangjun@zju.edu.cn>


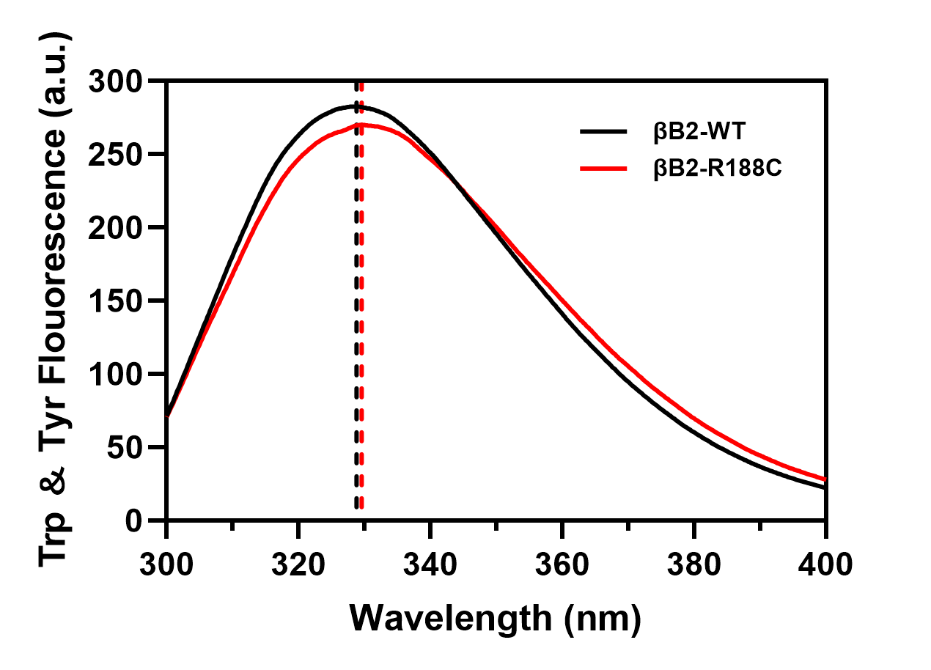


**Figure S1.** Intrinsic Trp and Tyr fluorescence spectra excited by 280 nm light.


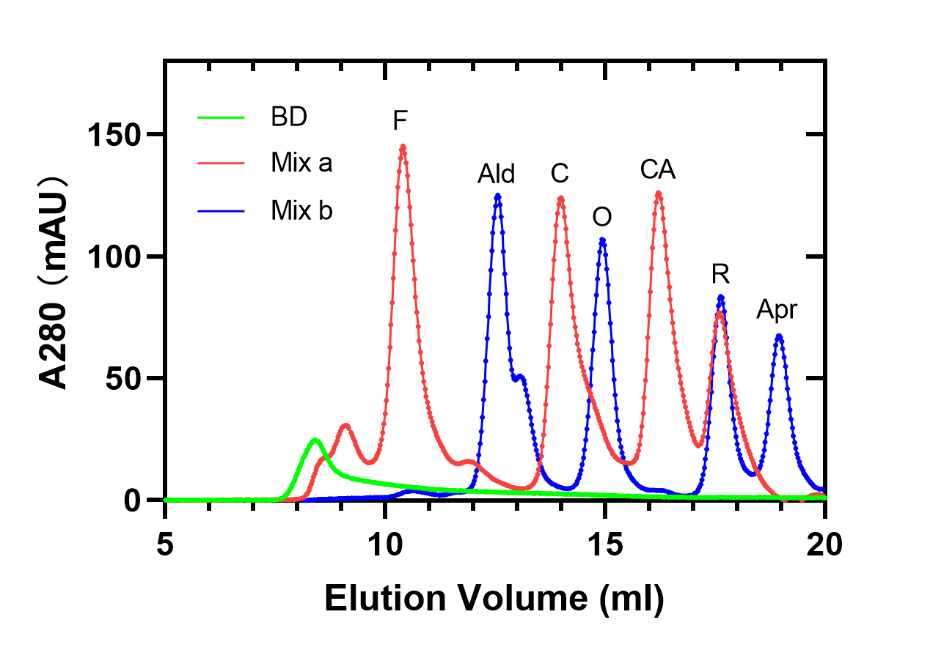


**Figure S2.** The calibration graph of the Superdex 200 10/300 GL column.


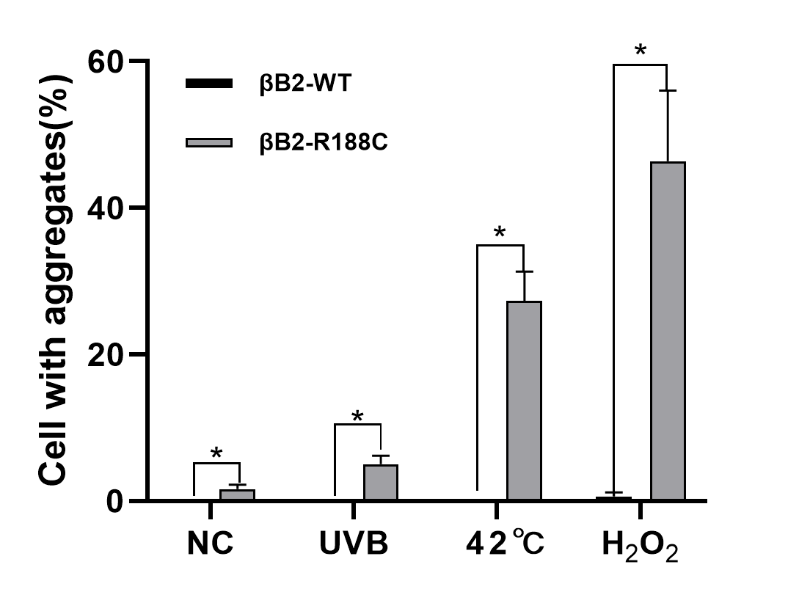


**Figure S3.** The proportion of cells with aggregates after normal culture, UV irradiation, high temperature and oxidative stress. n = 3, * *p* ＜ 0.05; ** *p* ＜ 0.01；*** *p* ＜ 0.001.


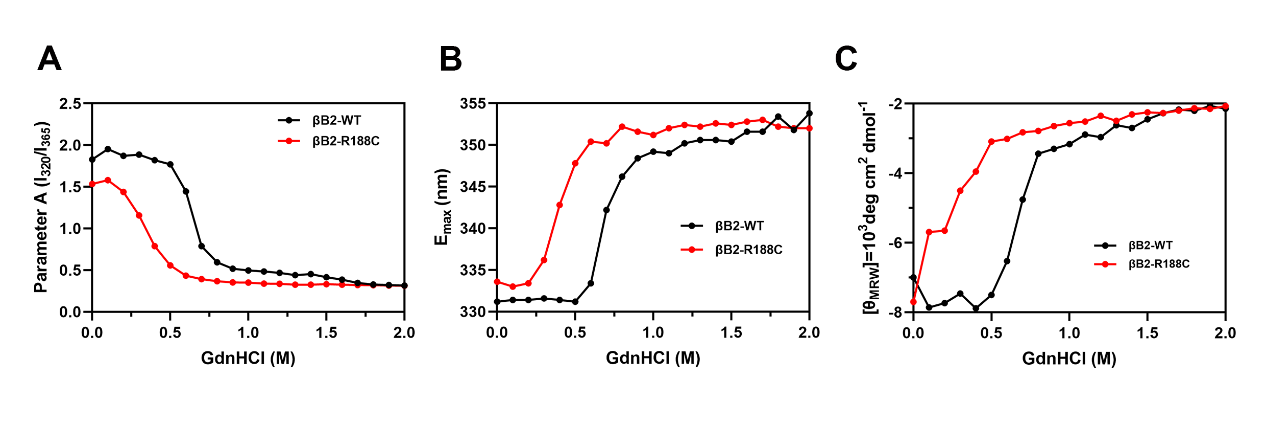


**Figure S4.** Effects of the R188C mutation on βB2-crystallin stability in guanidine hydrochloride (GdnHCl) solution. (A) Transition curves for parameter A, which is the ratio of fluorescence intensity at 320 nm to 365 nm. (B) Transition curves for E_max_ at Trp fluorescence elicited at 295 nm. (C) Transition curves overnight protein denaturation was plotted using ellipticities at 222 nm.


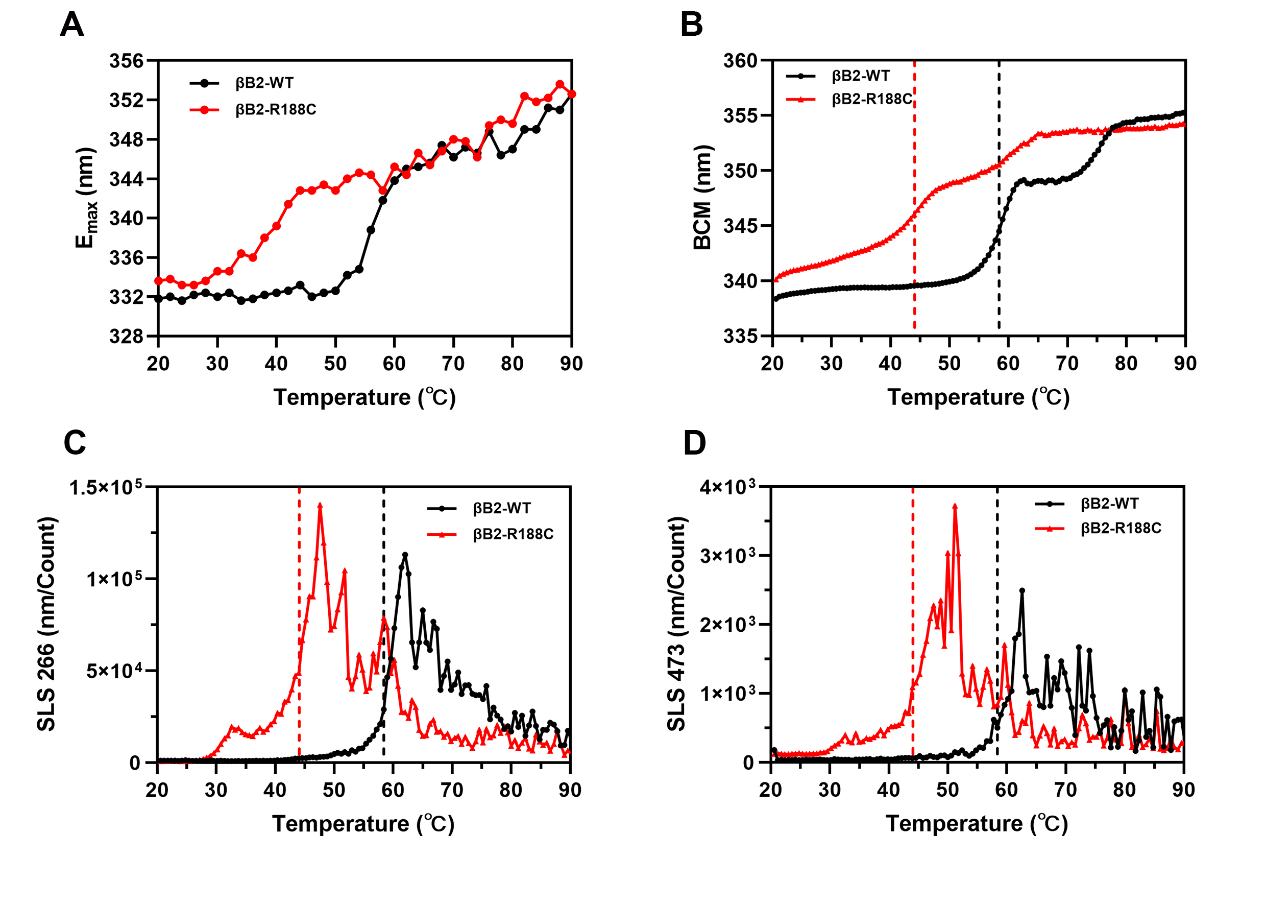


**Figure S5.** The thermal stability of βB2-WT and βB2-R188C. (A) Transition curves for E_max_ at Trp fluorescence elicited at 295 nm. (B) Transition curves for the barycentric mean of Trp fluorescence elicited at 295 nm. (C) Transition curves for static light scattering (SLS) at 266 nm. (D) Transition curves for SLS at 473 nm.


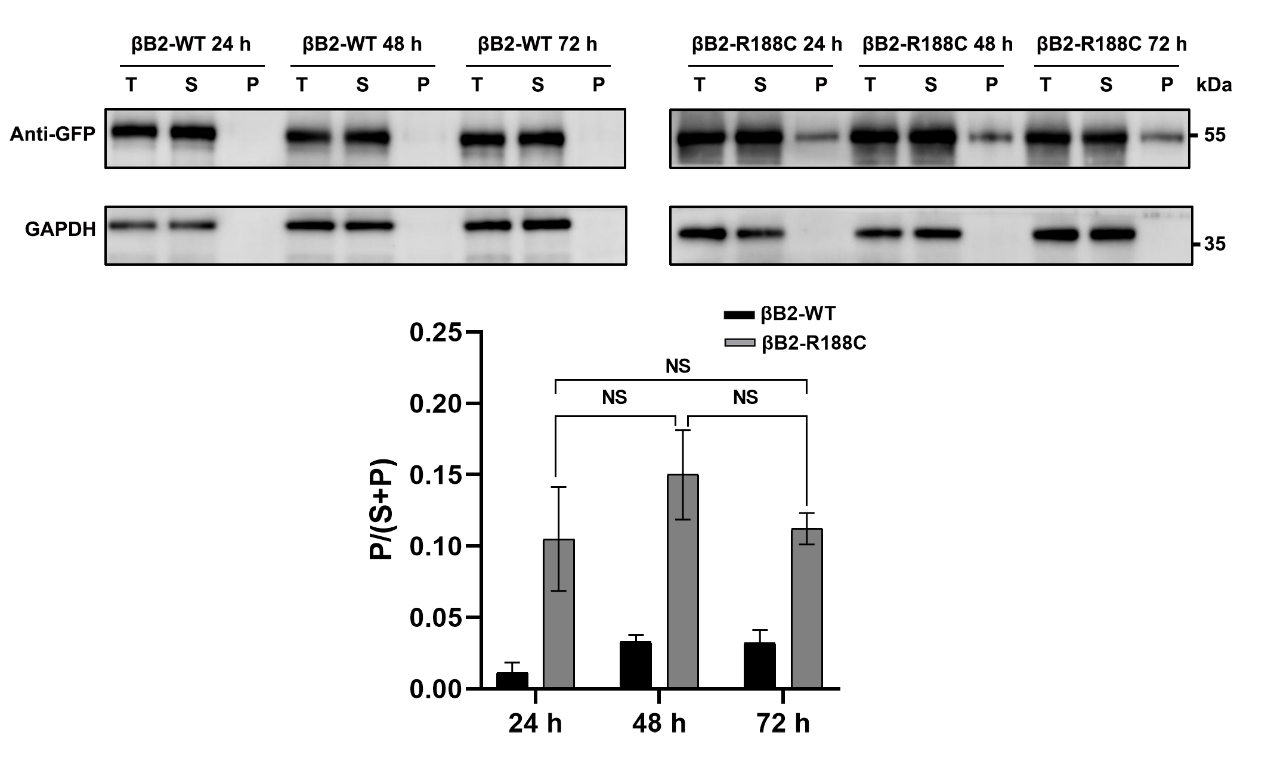


**Figure S6**. Abnormal protein aggregation trend of βB2-WT and βB2-R188C in HEK 293T cells at 24 hours, 48 hours and 72 hours. n = 3, NS = non significant.


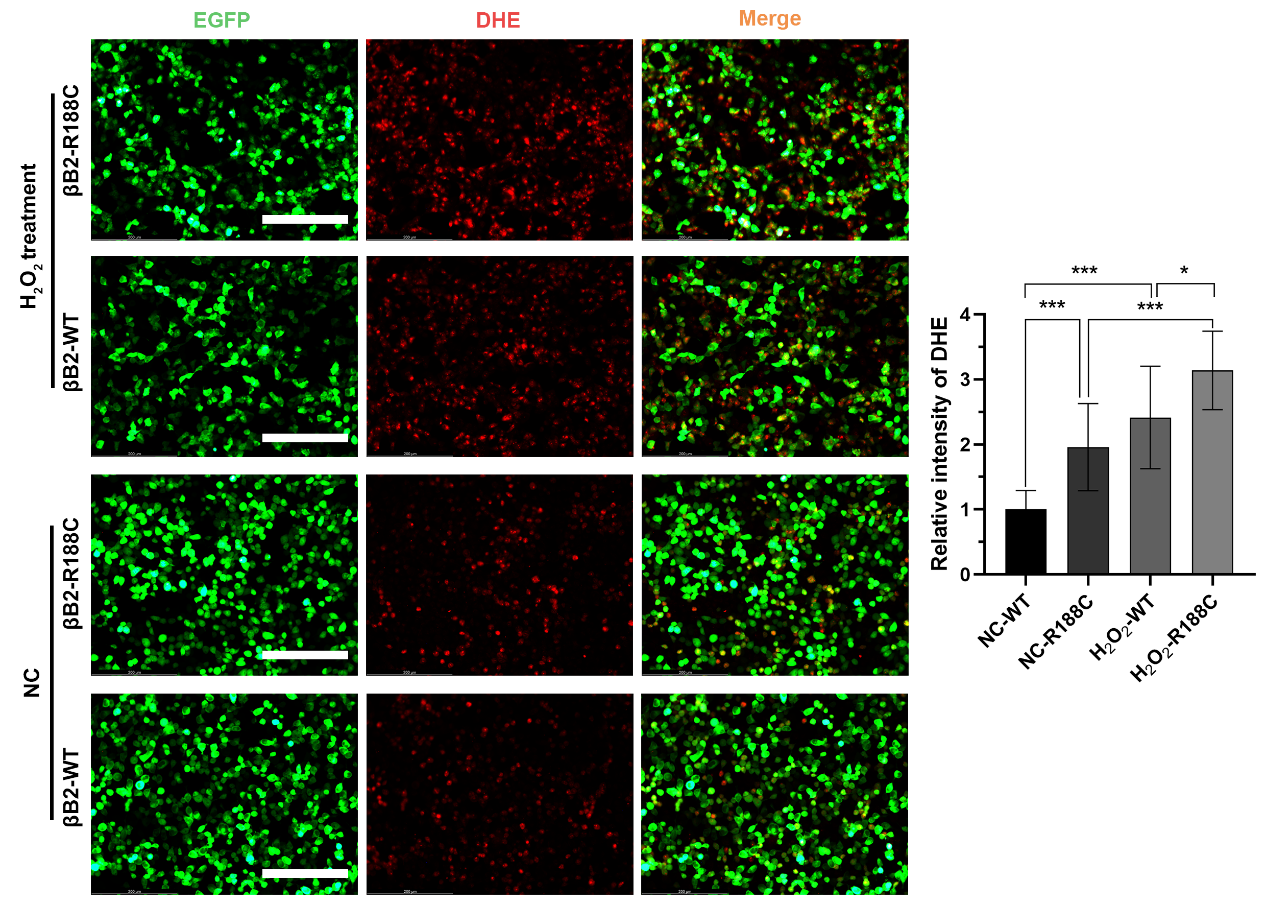


**Figure S7**. DHE staining and immunofluorescence quantitative analysis of HEK 293T cells in the four groups. Scale bars: 200 μm. n = 10, * means *p* < 0.05, ** means *p* < 0.01, *** means *p* < 0.001.


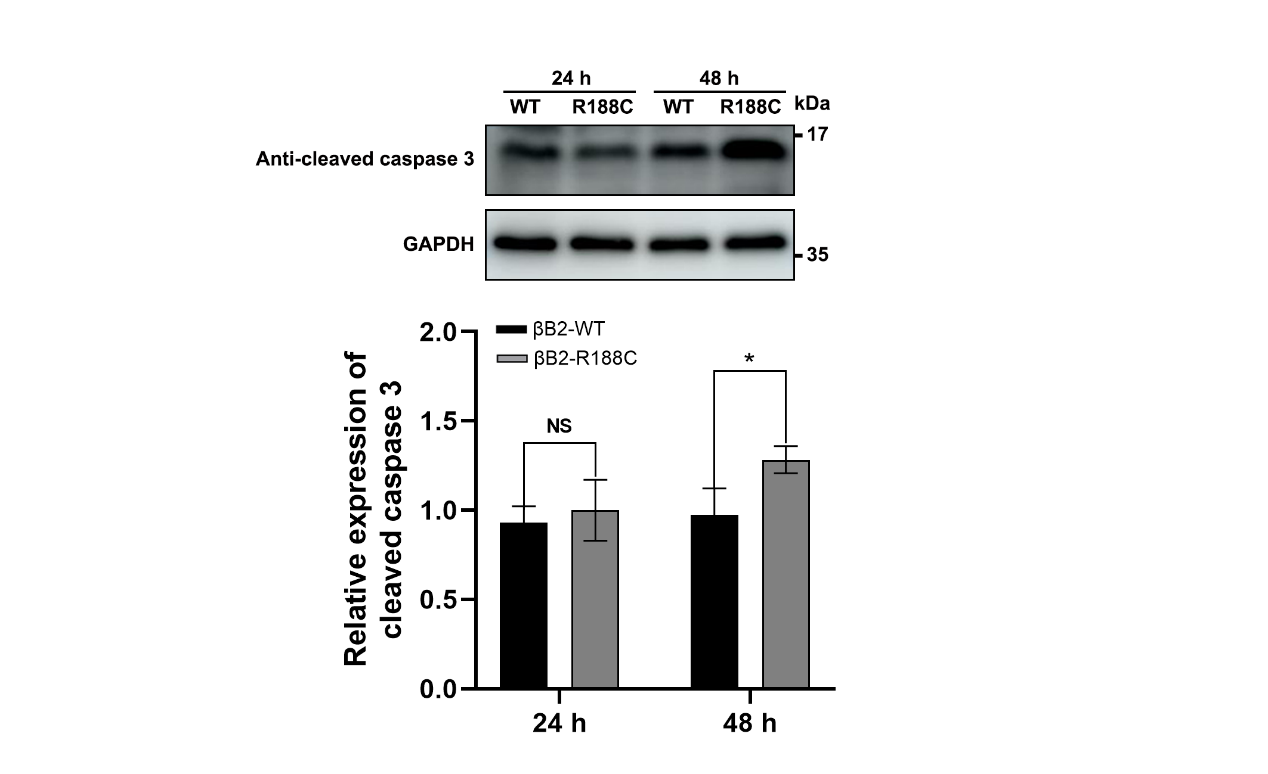


**Figure S8**. The expression of cleaved caspase 3 of two groups in 24 hours and 48 hours. n = 3, * means *p* < 0.05, NS = non significant.


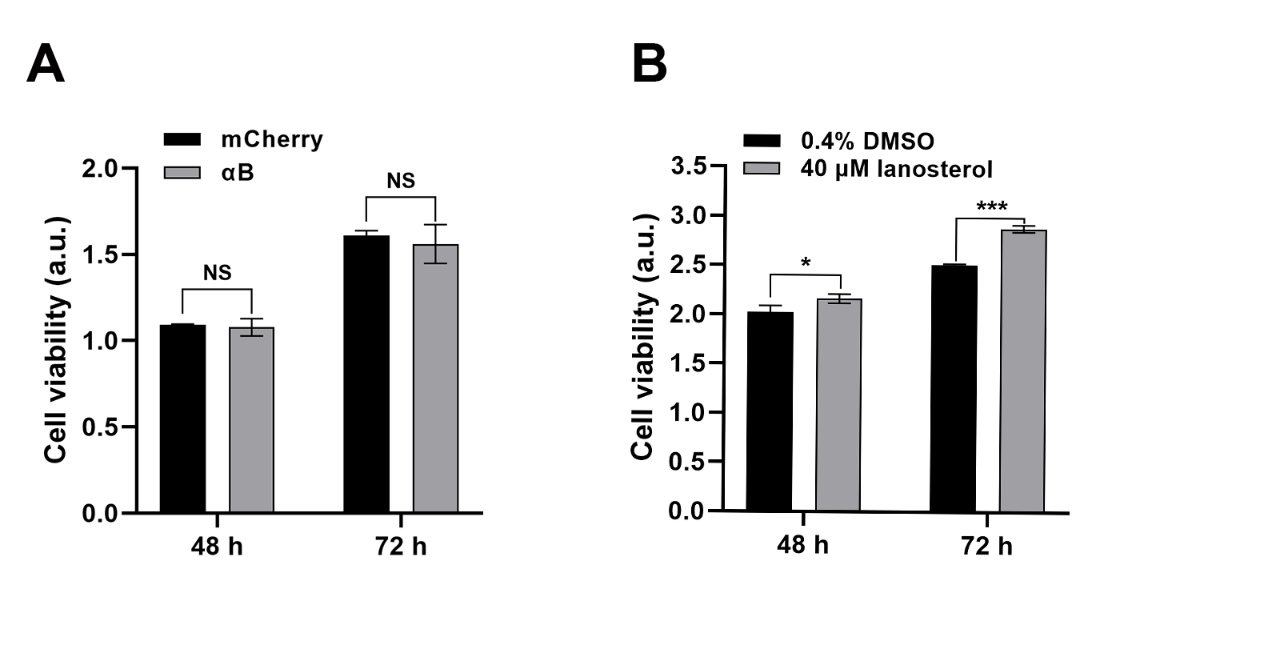


**Figure S9**. (A) Cell viability of HEK 293T cells after co-transfection of βB2-R188C and αB-crystallin plasmids at 48 hours and 72 hours. (B) Cell viability of R188C-overexpressed HEK 293T cells incubating with 40 μM lanosterol at 48 hours and 72 hours. n = 3, * means *p* < 0.05, ** means *p* < 0.01, *** means *p* < 0.001, NS = non significant.
